# Examining Interactions Between and Among Predictors of Net Ecosystem Exchange: A Machine Learning Approach in a Semi-arid Landscape

**DOI:** 10.1038/s41598-019-38639-y

**Published:** 2019-02-18

**Authors:** Qingtao Zhou, Aaron Fellows, Gerald N. Flerchinger, Alejandro N. Flores

**Affiliations:** 1 Department of Natural Resources, University of New Hampshire, Durham, NH USA; 2 USDA Agricultural Research Service Northwest Watershed Research Center, Boise, ID USA; 3 Department of Geosciences, Boise State University, Boise, ID USA

## Abstract

Net ecosystem exchange (NEE) is an essential climate indicator of the direction and magnitude of carbon dioxide (CO_2_) transfer between land surfaces and the atmosphere. Improved estimates of NEE can serve to better constrain spatiotemporal characteristics of terrestrial carbon fluxes, improve verification of land models, and advance monitoring of Earth’s terrestrial ecosystems. Spatiotemporal NEE information developed by combining ground-based flux tower observations and spatiotemporal remote sensing datasets are of potential value in benchmarking land models. We apply a machine learning approach (Random Forest (RF)) to develop spatiotemporally varying NEE estimates using observations from a flux tower and several variables that can potentially be retrieved from satellite data and are related to ecosystem dynamics. Specific variables in model development include a mixture of remotely sensed (fraction of photosynthetically active radiation (fPAR), Leaf Area Index (LAI)) and ground-based data (soil moisture, downward solar radiation, precipitation and mean air temperature) in a complex landscape of the Reynolds Creek Experimental Watershed (RCEW) in southwest Idaho, USA. Predicted results show good agreement with the observed data for the NEE (r^2^ = 0.87). We then validate the temporal pattern of the NEE generated by the RF model for two independent years at the two sites not used in the development of the model. The model development process revealed that the most important predictors include LAI, downward solar radiation, and soil moisture. This work provides a demonstration of the potential power of machine learning methods for combining a variety of observational datasets to create spatiotemporally extensive datasets for land model verification and benchmarking.

## Introduction

There is a clear need to develop additional approaches to map and understand carbon exchange at ecosystem-to-landscape scales in the northern Great Basin. Factors that impact carbon stocks and exchange are changing rapidly in this region. Many rangelands, for example, have experienced woody encroachment, cheatgrass invasion, and land use intensification that has altered the carbon cycle^1,2^. Climate change is expected to have additional widespread effects over the coming century^3^. Thus, considering the importance of arid and semiarid regions to the global carbon budget^4,5^ and the Great Basin to the Western US carbon cycle, there is a considerable need to develop an approach and examine patterns of Great Basin carbon cycling for both scientific research and management applications.

Filed measurements, modeling, and/or remote sensing are commonly combined to understand current and future carbon cycling at landscape-scales. In the northern Great Basin, there are a number of structural and functional ecological adaptations that make predicting Net Ecosystem CO_2_ Exchange (NEE) with existing process-based or remote sensing models challenging^6–8^. For example, important ecological structures such as plant functional types, microbial communities, soil depth, plant rooting depth, and water holding capacity are often poorly mapped. Key functional attributes such as leaf phenology linked to seasonal drought or rain, environmental cues for plant dormancy, and hydraulic redistribution are simply not well-represented in some current models, but are particularly important in Great Basin ecosystems^9^. Lastly, many factors that impact NEE are markedly non-linear, including the response of NEE to light, temperature, and water availability, and the mathematical parameterization and representation of these factors in models may not be well-constrained^10,11^. The sensitivity of plants to soil moisture availability, evaporative demand, and their interactions present a particular challenge. Commonly-used light use efficiency models, for example, represent plant water-stress in markedly different ways, with various combinations of vapor pressure deficit, warm air temperature, or soil moisture used as factors to limit NEE^12–14^.

Machine learning provides an alternative strategy to quantify NEE at local to global scales that is complimentary to existing approaches. Machine learning is an established approach in climatology and remote sensing fields^15,16^ and, more recently, has been applied to ecological and carbon cycle research^17,18^. Ideally, machine learning can produce predictive models without making specific assumptions about underlying ecological structure or the mathematical representation of key processes and interactions in an ecosystem. Specifically, machine learning can be used to identify key variables for predicting NEE, incorporate correlated and non-correlated variables, and side-step common modeling challenges such as equifinality or multi-colinearity. Thus, machine learning is particularly appealing due to its ability to combine field measurements with satellite imagery without making several prior assumptions, and possibility to improve with expanding field infrastructure, data management networks (e.g., Ameriflux), and remote sensing imagery. Although previous studies have used remote sensing data to estimate NEE at large scales, validation of estimates NEE for large scales are challenging because they are limited by the observational constrains. Our approach is unique in (1) exploring non-linear relationship between diverse variables and NEE (2) identifying those variables that are most influential in describing variation in NEE (3) providing insights into the drivers that affects carbon dynamics between land surface and atmosphere, and therefore constrain community land models that represent C dynamic.

In this study, we develop a random forest (RF) model, in a form of machine learning approach to predict the temporal variation of NEE in a semiarid ecosystem^19^. The main objectives are to conduct a case study to: (1) Develop predictive models of NEE using climate, soil, and vegetation variables for which there are either existing or planned remote sensing missions, field measurements, and a random forest model, (2) Verify the applicability of a random forest approach to estimate NEE in the cold and dry northern Great Basin, and (3) Understand the sensitivity of NEE predictions to uncertainties in the underlying biophysical predictor variables identified in model development. To accomplish these objectives, section 1 provides the background of NEE in Great Basin and RF model application, and summarizes the main objectives. Section 2 describes the study site, the sources of various datasets, development of RF model and model evaluation. Section 3 introduces results from linear regression analysis and RF model throughout the paper. Section 4 discusses model parameter uncertainties, predictors that might affect NEE and future researches on this topic. Section 5 summarizes major findings in this study.

## Methods

**Figure 1:**
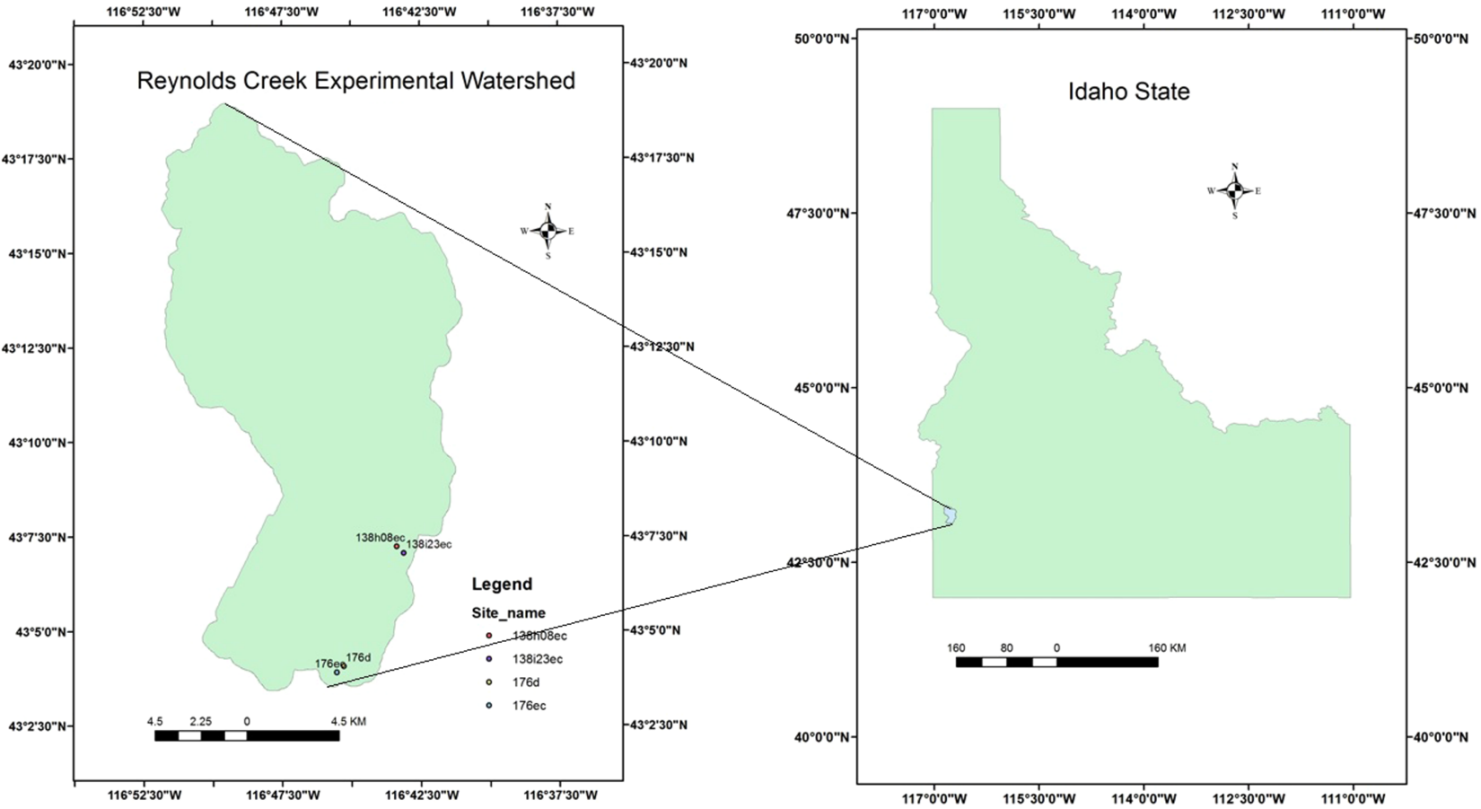
Reynolds Creek Experimental Watershed located in southwestern Idaho, USA, with two eddy flux towers. Note: the map is created by one of the authors using ESRI ArcGIS 10.4.1. http://desktop.arcgis.com/en/arcmap/.

The Reynolds Creek Experimental Watershed (RCEW) is a semiarid rangeland watershed with an area of 239 km^2^ located on the north flank of the Owyhee Mountains in Southwestern Idaho, USA (Fig. 1). This watershed was designated as a Critical Zone observatory funded by the National Science Foundation in 2014, and is operated by the U.S. Department of Agriculture’s Agricultural Research Service (ARS) Northwest Watershed Research Center (NWRC)^20–22^. It is characterized by a diversity of vegetation types, low precipitation, hot dry summers and mild wet winters. The dominant vegetation type is a patchwork of shrub-steppe, meadow, and bare ground with large stands of coniferous and deciduous forests^19^. The precipitation and climate vary greatly across the RCEW due to complex geophysical conditions. The mean annual precipitation across the RCEW is relatively low at lower elevations (200 mm/yr) than at higher elevations (>1100 mm/yr) (http://criticalzone.org/reynolds/about/). The mean annual temperature varies from 11 °C at lower elevations to 5 °C at higher elevations.

A variety of data sources were used, such as ground-based measurements for NEE and remote sensing datasets. Detailed information about how we obtained these sources of data are introduced in the following sections.

### Field Measurement for ecosystem carbon exchange

NEE was determined at four long-term eddy covariance sites (138h08, 138i23, 176 and 176d) in the Reynolds Creek Critical Zone Observatory using the eddy covariance approach. Ten-Hz carbon dioxide and water vapor concentrations were measured at ~2–5 m above the plant canopy using an open-path infrared gas analyzer mounted to scaffolding or a tower (LI-7500, LICOR Biosciences, Lincoln, Nebraska)^23^. Wind speed, wind direction, and sonic temperature were measured at 10-Hz with a three-dimensional sonic anemometer (CSAT3, Campbell Scientific, Logan, Utah) situated within 30 cm of the gas analyzer. We determined NEE at 30 min. intervals from the 10-Hz wind and trace gas observations using EddyPro software (EddyPro® version 5.2.1; LICOR Biosciences; Lincoln, Nebraska; https://www.licor.com). We selected the following processing options in EddyPro. Spikes and outliers in the 10-Hz data were removed with the software’s standard setting. Non-horizontal streamlines were rotated using the double rotation method. Deviations in the vertical wind speed and gas concentrations were determined using block averaging. The time-lags between the wind speed and gas concentration data is accounted for the maximum covariance between the time series. The air density effects on trace gas concentrations are also considered according to Webb *et al*.^24^. Fluxes were increased due to high-pass and low-pass filtering effects^25,26^. Missing observations in the NEE record were associated with instrument malfunction, non-turbulent conditions, and filtering on data quality. Non-turbulent NEE observations were identified with a friction velocity below 0.2 m/s.

These four sites (138h08, 138i23, 176 and 176d) within RCEW were evaluated for the current analysis. We chose sites 138h08 and 176d as representative of the four sites because they have longer data sets than the other two sites. In addition, sites 138h08 and 138i23 are in the same MODerate-resolution Imaging Spectroradiometer (MODIS) pixel, while sites 176 and 176d also share the same MODIS pixel. Sites 138h08 and 176d are characterized by different vegetation types, those being sagebrush and aspen, respectively; we refer to these sites as sagebrush and aspen rather than 138h08 and 176d. For the sagebrush site, the post-processed data were from August (DOY 229) 2005 to October (DOY 296) 2012. For this site, we used data from 2006–2012. For the aspen site, the data from 176d were only from February (DOY 44) 2007 to December (DOY 352) in 2012. Since sites 176 and 176d share the same MODIS pixel, we used the post-processed data from site 176 that are from February (DOY35) 2003 to December (DOY356) 2007 to fill the data gaps for site 176d. At the aspen site, we actually used data between 2004 and 2007 from site 176 and between 2008 and 2012 from site 176d.

### Meteorological and soil datasets

There are several sites in the RCEW that have long-term soil moisture data set measured hourly at 10 cm, 30 cm, 60 cm, 90 cm and 100 cm depths using a Steven Hydra Probe 2 (http://www.stevenswater.com/catalog/Stevens-Hydra-Probe.aspx). We used the hourly soil moisture data from site 138j09 within the sagebrush area near site 138h08. Site 176c, which is located within the same aspen grove as site 176d, provided hourly soil moisture data for the aspen site. Hourly soil moisture data were aggregated into daily soil moisture data for both sites. Hourly downward solar radiation and air temperature data were accessed through the ftp website, ftp://ftp.nwrc.ars.usda.gov/. The hourly downward solar radiation data were then aggregated to daily data. Downward solar radiation was measured with Eppley precision spectral pyranometers, which were sensitive to wavelengths from 285–2800 nm and the data were expressed in terms of W/m^2^. The mean daily air temperature was obtained by averaging the daily maximum and minimum temperatures.

### Remote sensing datasets

We obtained Leaf Area Index (LAI), and fraction of Photosynthetically Active Radiation (fPAR) data derived from the MODerate-resolution Imaging Spectroradiometer (MODIS); (MCD15A3) for the Reynolds Creek Experimental Watershed from 2000–2013. We used a 1-km-resolution, 4-day composite MODIS product (MCD15A3). Two quality control layers are contained in the product. To obtain the daily LAI and fPAR data, we used an interpolation and smoothing method similar to that of Dozier *et al*. (2008) and applied an interpolation and smoothing algorithm using Matlab (version MatlabR 2015) to fill the temporal data gaps along the time axis (f(t)). The smoothing algorithm is conducted to obtain estimates for f(t). The smoothing spline function is as follows:

1 $${\rm{f}}({\rm{t}})={\rm{p}}\sum _{{\rm{j}}={\rm{1}}}^{{\rm{N}}}\,{\rm{w}}({\rm{j}}){|\hat{{\rm{f}}}({{\rm{t}}}_{{\rm{j}}})-{\rm{f}}({{\rm{t}}}_{{\rm{j}}})|}^{2}+(1-p){\int }_{{t}_{min}}^{{t}_{max}}\,\lambda (t){|{D}^{2}f(t)|}^{2}dt$$

where w(j) is a weight vector and the default value in the error measure is one (size(x)). *λ*(*t*) is w weight function and the default value is 1. D^2^f(t) is the second derivative of the function f(t) and *p* is a smoothing parameter, varying between 0 and 1. If p = 0, f(t) is the least-squares straight line fit to the data. More detailed information can be read from the website http://www.mathworks.com/help/curvefit/csaps.html.

### The RF model

We used the random forest model available within Matlab, together with the classification and regression training package (CARET), to test several machine learning algorithms present in CARET. Matlab is sufficient for data analysis and has its own high quality machine learning algorithm for users - CARET. The RF method is one of many machine learning approaches. It is an ensemble classifier that consists of many decision trees^27^. Decision trees predict the dependent variable or target based on different independent variables or attributes. Each decision tree is built by best splitting the data by searching through the random attributes. We will use a dataset (N represents all the predictors) as an example. Then the next step is to grow the decision tree. For each decision tree, there is a test in each node to test the value of an attribute and compare it with a constant. m (m < N) variables are randomly selected from all the predictors (N). Then the predictor variables that provide the best split, based on some comparisons, is used to do a binary split on that node. Then at the next node, choose another m variables at random from all predictor variables and do the same. The decision tree is grown until it reaches its fullest when there is no split. The data that are not used to develop the random forest model are “out of bag” samples. Out of bag samples are used to unbiasedly test the model based on the training dataset.

After we use the RF method to build a model, we need to evaluate how well the model works. Evaluating the important predictors is a crucial step for using the RF model. The model is favorable for using more important predictors than less important predictors. In our study, we used Matlab to apply an existing algorithm that can be used to predict important variables to explore the important predictors (http://www.mathworks.com/help/stats/compactregressionensemble.predictorimportance.html). The metric for assessing the importance for each predictor is calculated by summing changes in the mean squared errors due to splits on every predictor and then dividing the sum by the number of branch nodes. A higher value indicates that the predictor is more important.

### Model evaluation

We divided our available data into two subsets: a training set and a validation set. The daily predictors from 2005 to 2012 except 2008 and 2009 were used as the training data set and the data from 2008 and 2009 were the validation data set. The random forest model was built based on the training dataset. Then the model was used to predict NEE data for 2008 and 2009 based on the predictors in these two years. The ground based measurements for 2008 and 2009 were therefore used to validate the model. Besides this, we also developed an algorithm that can randomly select unknown numbers of predictors ($${{\rm{C}}}_{6}^{1}+{{\rm{C}}}_{6}^{2}+{{\rm{C}}}_{6}^{3}+{{\rm{C}}}_{6}^{4}+{{\rm{C}}}_{6}^{5}+{{\rm{C}}}_{6}^{6}=$$$$6+15+20+15+6+1=63$$). Accordingly, we run the model 63 times and calculate the error metrics for the 63 combinations.

Daily NEE output from the random forest model for the two sites from 2005 to 2012 based on the input daily predictors were evaluated using several steps. The first step was to use a regression fit to check the agreement between prediction data and ground based measurements. A linear regression of modeled vs. derived values was fitted to calculate the coefficient of determination (r^2^), which was used to decide if the fit was good. The second step was to calculate statistical metrics comparing simulation results and ground based measurements. Several statistical indices were considered to evaluate model performances and are calculated as follows:

2 $$RMSE=\sqrt{\frac{{\sum }_{i=1}^{n}\,{({X}_{obs,i}-{X}_{pre,i})}^{2}}{n}}$$

3 $$BIAS=\frac{1}{n}\,\sum _{i=1}^{n}\,({X}_{pre,i}-{X}_{obs,i})$$

4 $$MAE=\frac{1}{n}\,\sum _{i=1}^{n}\,|{X}_{obs,i}-{X}_{pre,i}|$$

5 $$MAPE=\frac{100}{n}\,\sum _{i=1}^{n}\,|\frac{{X}_{obs,i}-{X}_{pre,i}}{{X}_{obs,i}}|$$

where ${{\rm{X}}}_{{\rm{pre}},{\rm{i}}}$ is the predicted value at time t; ${{\rm{X}}}_{{\rm{obs}},{\rm{i}}}$ is the observed value at time t; n is the number of observations.

## Results

### Temporally distributed NEE, fPAR, LAI, mean air temperature and soil moisture at the sagebrush and aspen sites

Ground-based NEE measurements from the sagebrush and aspen sites show a distinct seasonal trend. Both sites show higher NEE values in winter and lower values in summer, with a maximum of 0.55 mg C/m^2^ day^−1^ and 0.14 mg C/m^2^ day^−1^ and a minimum of −2.46 mg C/m^2^ day^−1^ and −2.84 mg C/m^2^ day^−1^ at the sagebrush and aspen sites, respectively. Both sites exhibit net CO_2_ gains in summer (mean + standard deviation: −1.37 ± 0.64 mg C/m^2^ day^−1^ and −2.26 ± 0.50 mg C/m^2^ day^−1^, respectively). The sagebrush site shows a net CO_2_ loss in winter (0.22 ± 0.085 mg C/m^2^ day^−1^), while the aspen site shows a net gain in winter (−0.058 ± 0.099 mg C/m^2^ day^−1^). The aspen site is a CO_2_ sink for a much longer period (216 days) compared to the sagebrush site (140 days). Sagebrush site is a CO_2_ source for a much longer period (225 days) compared to the aspen site (149 days). The timing of the peak negative NEE occurs later at the aspen site (DOY 201) than the sagebrush site (DOY 171).

**Figure 2:**
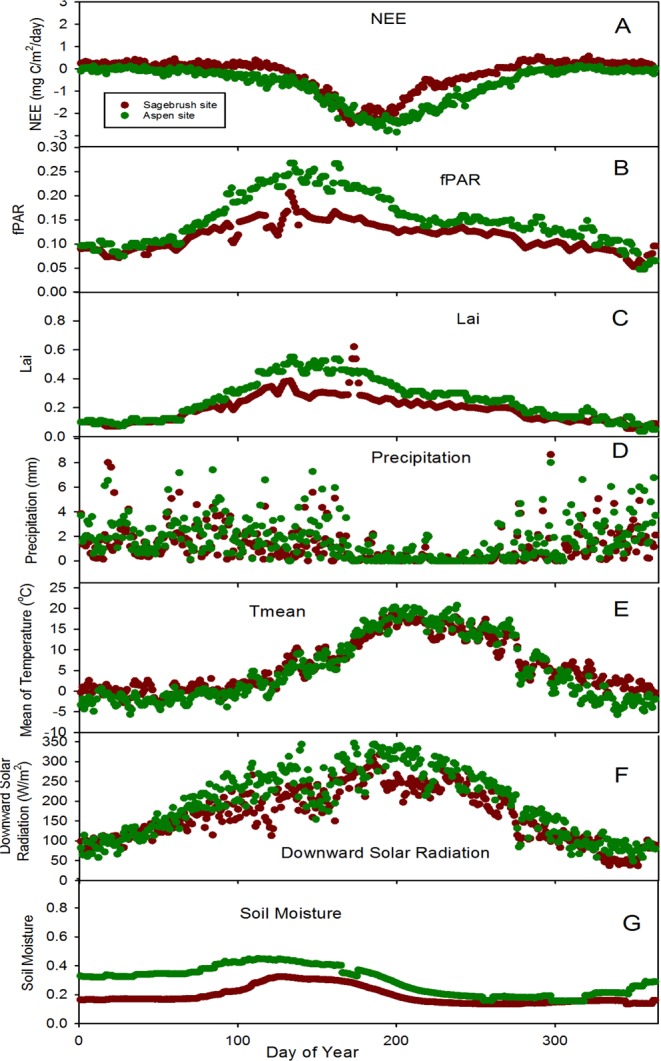
The daily average for the NEE (**A**), fPAR (**B**), LAI (**C**), precipitation (**D**), mean air temperature (**E**), downward solar radiation (**F**), and soil moisture (**G**) between 2006–2012 for the sagebrush site, and between 2005–2012 for the aspen site based on ground based measurements. The mean air temperature is obtained by averaging the maximum and minimum temperatures for the day. Soil moisture data are the average of five different soil depths.

Remote sensing data for fPAR and LAI show similar patterns (Fig. 2B,C). The values for fPAR and LAI are low in autumn and winter and high in spring and summer. They increase from spring to summer due to vegetation growth and then decrease in autumn and winter. The range for fPAR at the sagebrush and aspen sites are 0.05–0.21 and 0.05–0.27, and for LAI are 0.057–0.45 and 0.038–0.54, respectively (Fig. 2B,C). The fPAR and LAI estimates are generally higher at the aspen site than at the sagebrush site.

Daily precipitation at the two sites is low in summer (mean 0.51 ± 0.9 mm and 0.71 ± 1.2 mm at the sagebrush and aspen sites, respectively) and high in winter (mean 1.8 ± 1.5 mm and 2.5 ± 1.5 mm), spring (mean 1.6 ± 1.3 mm and 2.4 ± 1.6 mm), and autumn (mean 0.9 ± 1.4 mm and 1.4 ± 1.5 mm). The highest precipitation occurs during the winter. The precipitation at the aspen site is relatively higher than at the sagebrush site in winter, spring, summer and autumn.

The two sites show a wide range of mean daily air temperature (18.7 °C to −4.0 °C, 20.4 °C to −5.7 °C) at the sagebrush and aspen sites respectively based on the long-term data. The mean temperature at the aspen site is higher than at the sagebrush site in summer (15.0 ± 4.3 °C and 13.9 ± 3.5 °C, respectively), while it is lower than at the sagebrush site in spring (1.5 ± 3.2 °C and 2.7 ± 3.0 °C, respectively), autumn (6.7 ± 6.6 °C and 7.8 ± 4.6 °C) and winter (−2.4 ± 1.4 °C and 0.3 ± 1.6 °C).

The temporal variability for the downward solar radiation flux ranges from 346 W/m^2^ to 49 W/m^2^ and from 347 W/m^2^ to 58 W/m^2^ at the sagebrush and aspen sites, respectively. The aspen site, being at higher elevation, has slightly higher downward solar radiation than the sagebrush site.

The temporal soil moisture patterns are similar at the two sites. They are high in early spring and low in summer, autumn and winter. The highest soil moisture occurs in early summer and the lowest soil moisture occurs in early autumn. Specifically, temporal daily soil moisture ranges from 0.14 to 0.32, and 0.15 to 0.45 at the sagebrush and aspen sites, respectively. Soil moisture at the aspen site is higher than at the sagebrush site.

### Exploring relationships between the NEE and different single factors

**Figure 3:**
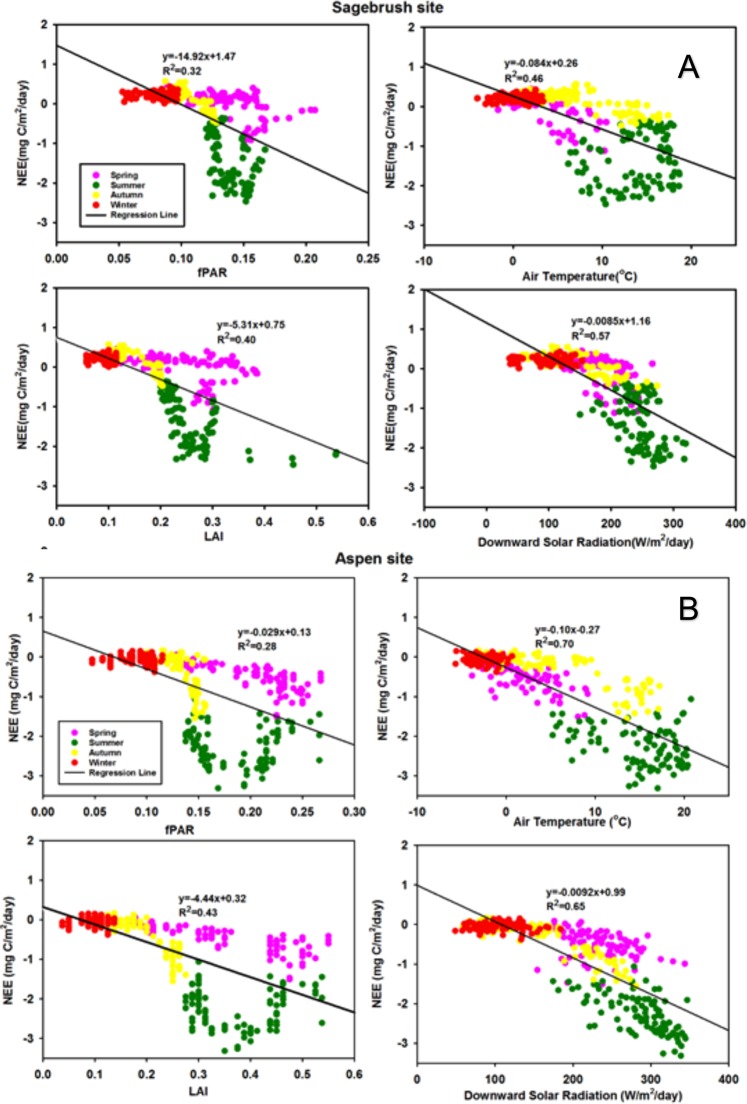
The relationship of the fPAR, LAI, precipitation, mean air temperature, downward solar radiation, and soil moisture with the NEE based on data for 2005–2012 and 2006–2012 for the sagebrush (**A**) and aspen sites (**B**), respectively. Pink, green, yellow and red colors represent spring, summer, autumn and winter, respectively.

We explored the linear regression relationship between the daily NEE and fPAR, LAI, mean air temperature and soil moisture at the sagebrush and aspen sites. NEE has strong relationships with LAI (r^2^ = 0.40 and r^2^ = 0.43), mean air temperature (r^2^ = 0.46 and r^2^ = 0.70), and downward solar radiation (r^2^ = 0.57 and r^2^ = 0.65) at the two sites, respectively (Fig. 3). There is no strong linear relationship between the NEE and precipitation (r^2^ = 0.08 and r^2^ = 0.19), and soil moisture (r^2^ = 0.18 and r^2^ = 0.0011).

Figure 3 suggests some linear relationships between the NEE and the parameters described above. However, simply using one or two parameters based on a linear regression relationship cannot reflect the real temporal pattern of the NEE. There may be some non-linear information included in these parameters. Therefore, it is important to explore non-linear methods, such as Random Forest models, to pursue more accurate simulation results for the NEE.

### Validation of results

**Figure 4:**
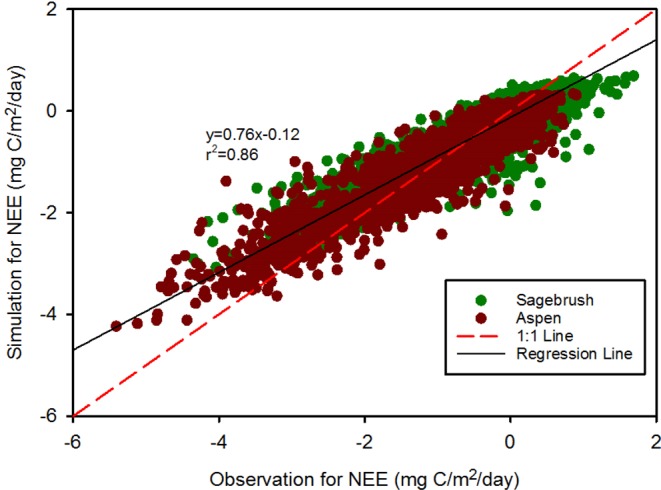
NEE predictions based on the RF model compared to ground-based data during the training period. The solid black line is the 1:1 line and the dotted red line is the regression line.

Overall, the agreement between the simulation and observation data is good for the training dataset (r^2^ = 0.87) (Fig. 4). The mean simulation NEE (−0.24 ± 0.70 mg C/m^2^ day^−1^) is similar to the mean observed data (−0.24 ± 0.54 mg C/m^2^ day^−1^). The simulation data are from −2.80 mg C/m^2^ day^−1^ to 0.66 mg C/m^2^ day^−1^, while the observed data are from −4.8 mg C/m^2^ day^−1^ to 1.6 mg C/m^2^ day^−1^. The RF model also shows that the three most important factors are LAI, downward solar radiation and soil moisture (Table 1).

**Table 1:** Importance of daily meteorological variables in predicting net ecosystem exchange (NEE) based on the Random Forest (RF) model.

Predictors	Importance of predictors
fPAR	1.091
LAI	1.673
Precipitation	0.205
Mean Air Temperature	1.599
Downward Solar Radiation	1.674
Soil Moisture	1.665

**Figure 5:**
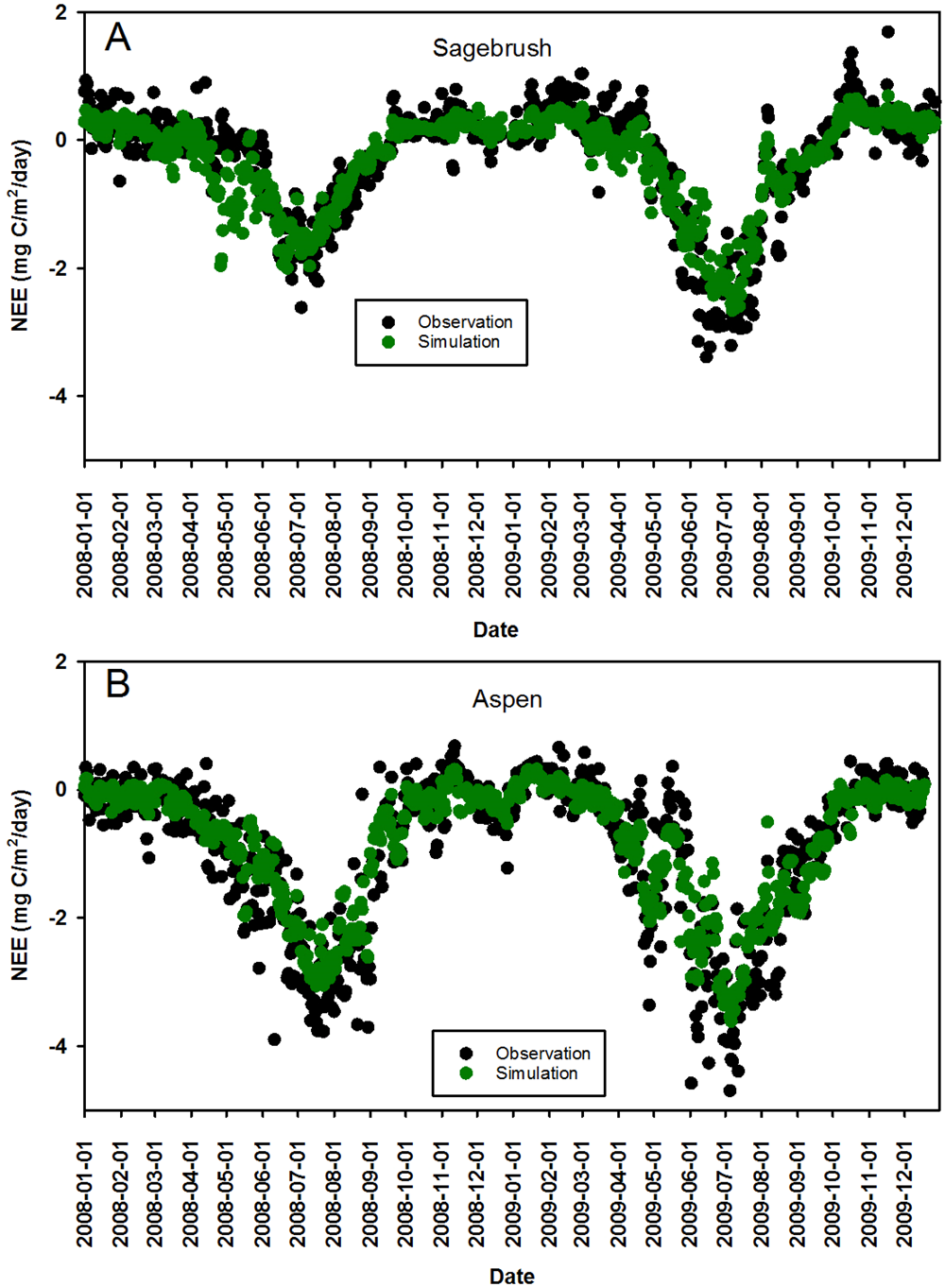
Validation for the sagebrush and aspen sites between 2008 and 2009 based on the RF model built on data for 2006–2012, excluding 2008–2009, for the sagebrush site, combined with data for 2005–2012, excluding 2008–2009, for the aspen site.

**Figure 6:**
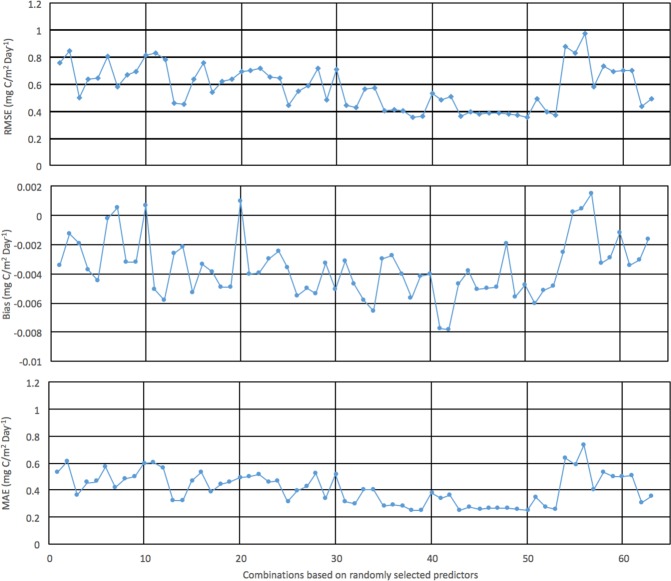
RMSE, Bias, MAE for all possible combinations based on RF model runs.

We validated the simulation results at the two sites with ground based measurements between 2008 and 2009 not used in the training dataset (Fig. 5). Generally, the simulation data captured the daily NEE pattern for these two years. The simulation data match well with the observed data. NEE is relatively stable in spring, and starts decreasing in late spring and summer. Then NEE increases in the mid-summer and autumn. In late autumn and winter, NEE does not change. In summer, the model under-predicts the observed data, which is shown in Fig. 4. We also evaluate the error matrices (RMSE, Bias, and MAE) for all the possible combination (n = 63) using the model. RMSE, Bias and MAE range from 0.36–0.98 mg C/m^2^ day^−1^, −0.007–0.002 mg C/m^2^ day^−1^ and 0.26–0.73 mg C/m^2^ day^−1^ respectively (Fig. 6). Peak values for the three matrices occurred in the same time period.

## Discussion

As mentioned earlier, NEE has significant linear relationships with LAI, fPAR, air temperature and downward solar radiation (Fig. 3). Based on the study of Williams and Rastetter^28^ on arctic terrestrial ecosystems, NEE is correlated with LAI and the correlation can help constrain model parameters. This is consistent with our study in the RCEW. LAI data can reflect the characteristics of the land surface. fPAR and LAI deduced from MODIS are relatively low compared with the observed data from the RCEW (Fig. 2), which is attributed to errors from MODIS products and field experiments. Some studies have shown that the retrieved LAI from MODIS products are always underestimated due to MODIS LAI algorithm. The underestimation error becomes larger as spatial resolution decreases or heterogeneity increases^29^.

It is commonly known that NEE has a strong relationship with mean air temperature and light, which are widely used for NEE gap filling methods. Gilmanov *et al*.^10^ showed that a semi-empirical model based on a light-temperature response can provide good predictability for estimating photosynthesis and respiration. Some other studies argued that this light-temperature response model may not be applicable for some water-stressed ecosystems like semi-arid environments, as shown by Kwon *et al*.^30^ for south central Wyoming. However, our study is consistent with the results reported by Gilmanov *et al*.^10^. The ground-based downward solar radiation measurements are strongly related to the light conditions.

In addition, the relationship between NEE and fPAR, LAI, mean air temperature, and soil moisture, shows strong seasonal patterns (Fig. 2). Both fPAR and LAI increase in spring, then decline in summer. These vegetation parameters reach the lowest turning point for NEE in summer, which suggests CO_2_ uptake for this semi-arid ecosystem is at its maximum at this time period due to photosynthesis. CO_2_ sink decreases in autumn and the ecosystem changes from a CO_2_ sink to a CO_2_ source. In winter, NEE shows positive values due to more CO_2_ released into the atmosphere. Mean air temperature and downward solar radiation show strong negative relationships with NEE.

Soil moisture is one of the most important factors based on the RF model (Table 1). It is also the most uncertain factor for a number of reasons. Soil moisture varies greatly depending on the location where the measurements are taken and is therefore limited by the measurement locations. Water is needed for plant growth, but if the vegetation experiences water stress, water availability may limit growth. Thus, soil moisture is a very important factor in an ecosystem where water is limited. Kwon *et al*.^30^ found that soil moisture (at soil depth 15–45 cm) has a strong relationship with the daytime and nighttime NEE during the growing season. Our study does not show any significant linear relationship between soil moisture and NEE (Fig. 3A,B) based on ground based measurements. However, the RF model shows that soil moisture is an important predictor for estimating NEE of CO_2_. In this study, we used average daily soil moisture storage for the entire soil profile, not just for a single soil depth for a particular period such as growing season. Simple linear regression analysis is not adequate to explore the relationship between different factors and the NEE.

Soil moisture exhibits different characteristics at the sagebrush site compared to the aspen site. At the aspen site, the soil moisture stays relatively consistent throughout all seasons. At the sagebrush site, soil moisture shows seasonal patterns. During early spring, precipitation and snowmelt increases the soil moisture. It continues to increase until summer. During summer, soil moisture decreases because of increased evapotranspiration and reduced precipitation. The aspen site has relatively higher soil moisture than at the sagebrush site because of the increased soil water input and subsurface lateral flow from the snow drifts that accumulate near the aspen groves^20^. The different soil moisture characteristics may be due to the different types of vegetation at the two sites.

As mentioned earlier, the NEE simulation captured the seasonal patterns for observed NEE values. Only during the summer did the NEE simulation overestimate the observed NEE values. There are two possible reasons for this. The first reason is that nighttime respiration, dissolved organic carbon and filtering errors contributes to the uncertainty of NEE for the field- based measurement. Nighttime respiration is subject to uncertainty due to geological and climatic factors^31^. During the daytime, respiration may be well recorded. However, during the night, respiration may not be well recorded and underestimated due to low wind velocity, rough geology (i.e., sloping mountainous terrain), and extreme weather^32^. Filter errors may add to the uncertainty in NEE estimates, due to temperature, incoming solar radiation and wind speed^32^. After obtaining the raw data from eddy flux tower, the flux data are first processed over 30 minute intervals and then completed with quality assurance. However, during the processing procedure, high temperature measurements may expand the CO_2_ volume, and cause a decreased CO_2_ density, which will underestimate the NEE of CO_2_.

The second reason is the LAI and fPAR data from MODIS products. LAI is always underestimated by the MODIS non-linear algorithm^33^. Additionally, uncertainties involved in the retrieval of LAI and fPAR can be related to (1) geographic characteristics of the sites, (2) atmospheric corrections, and (3) view-angle effects. The study area is a mountainous region, which can be a source of bias in MODIS at two stages. During the sensor’s overpass, initial local effects, such as complex terrain, may cause more uncertainties in the observations. Then, when the smoothing and interpolation method is applied to obtain the LAI and fPAR data, noise is eliminated to obtain smoothed data, which may lose some data characteristics.

Although model results have shown the model is robust and model performance is generally good for the training and testing datasets (Figs 4 and 5; Table 2), there are still potential limitations for the RF model. First we do not specifically point out which model would produce the best model performance. All the possible combinations of the model simulations are run to obtain objective evaluation for the model. Future study could focus more on choosing the best mole based on the model error matrices. Second, we do not consider the land use/change in the model. The study conducted by Ueyama *et al*. has shown that the upscaled CO_2_ fluxes in Alaska was relevant with the land use change information. Our model can incorporate land use/change information to examine the impacts of land use/change on NEE. Third, our study focus on temporal scale for NEE and do not consider the spatial patterns. To achieve this, obtaining the spatiotemporally distributed predictors for NEE is critical. Several potential satellite product datasets could be incorporated into the study such as Global Precipitation Measurement (GPM. http://www.nasa.gov/mission_pages/GPM/main/index.html) and Soil Moisture Active Passive (SMAP. http://smap.jpl.nasa.gov/). GPM can provide long term datasets for rain and snow and SMAP can provide valuable soil datasets. If potential predictors (e.g., soil moisture, precipitation etc.) could be retrieved from satellite products and were validated well with the ground-based measurements in RCEW, the spatially distributed soil moisture data across RCEW would be used to develop the spatiotemporal NEE across RCEW.

**Table 2:** Statistical indices for evaluating model errors in the two sites.

Sites	RMSE	BIAS	MAE	MAPE
Sagebrush	0.39	0.03	0.26	2.65
Aspen	0.45	−0.09	0.31	1.39

Note: the units for RMSE, BIAS and MAE are mg C/m^2^ day^−1^. The unit MAPE is 100%.

## Conclusions

This study of NEE of CO_2_ was carried out at two sites (sagebrush and aspen site) at Reynolds Creek Experimental watershed in southwestern Idaho. We used a machine learning approach combining remotely-sensed and ground-based data to capture the temporal pattern of NEE of CO_2_ from 2006 to 2012 or 2005 to 2012 at these two sites. The results showed that the random forest (RF) model can be effectively used with predictors from MODIS product to predict NEE of CO_2_. Moreover, this study explored the non-linear relationship between NEE of CO_2_ and different predictors. It showed that the most important predictors for NEE of CO_2_ are downward solar radiation, leaf area index, and soil moisture. Future study can focus on potentially using satellite products based on present methods.

## Data Availability

The data can be publicly accessed through the following website: ftp://ftp.nwrc.ars.usda.gov/reynolds-creek-datasets.
